# A Saccade Based Framework for Real-Time Motion Segmentation Using Event Based Vision Sensors

**DOI:** 10.3389/fnins.2017.00083

**Published:** 2017-03-03

**Authors:** Abhishek Mishra, Rohan Ghosh, Jose C. Principe, Nitish V. Thakor, Sunil L. Kukreja

**Affiliations:** ^1^Singapore Institute for Neurotechnology, National University of SingaporeSingapore, Singapore; ^2^Department of Electrical and Computer Engineering, University of FloridaGainesville, FL, USA; ^3^Biomedical Engineering Department, Johns Hopkins UniversityBaltimore, MD, USA

**Keywords:** motion segmentation, dynamic vision sensors, asynchronous signal processing, temporal information, tracking and following, robotics

## Abstract

Motion segmentation is a critical pre-processing step for autonomous robotic systems to facilitate tracking of moving objects in cluttered environments. Event based sensors are low power analog devices that represent a scene by means of asynchronous information updates of only the dynamic details at high temporal resolution and, hence, require significantly less calculations. However, motion segmentation using spatiotemporal data is a challenging task due to data asynchrony. Prior approaches for object tracking using neuromorphic sensors perform well while the sensor is static or a known model of the object to be followed is available. To address these limitations, in this paper we develop a technique for generalized motion segmentation based on spatial statistics across time frames. First, we create micromotion on the platform to facilitate the separation of static and dynamic elements of a scene, inspired by human saccadic eye movements. Second, we introduce the concept of *spike-groups* as a methodology to partition spatio-temporal event groups, which facilitates computation of scene statistics and characterize objects in it. Experimental results show that our algorithm is able to classify dynamic objects with a moving camera with maximum accuracy of 92%.

## 1. Introduction

Motion segmentation is an important task for applications that involve a moving camera or neuromorphic sensor, particularly in the field of robotics. However, most of the literature has addressed the identification of moving objects in a scene against a static background, which is an easier task. When standard frame-rate cameras are employed, the difference between image frames is the simplest method to detect static or dynamic events (Sobral and Vacavant, 2014). Another technique is to calculate optical flow vectors from consecutive frames to estimate regions of coherent motion (Narayana et al., 2013). Graphical models are also effective since they provide a simple yet efficient segmentation method (Badrinarayanan et al., 2013). Standard cameras have intrinsic limitations associated with uneven illumination, computations on each pixel in a frame, blurring of moving objects and limited frame-rate. Weinland et al. (2011) provide a comprehensive survey of strategies used for motion segmentation of dynamic objects using traditional image sensors.

Event based sensors (i.e., neuromorphic sensors) such as the dynamic vision sensor (DVS) (Lichtsteiner et al., 2008) and asynchronous time-based image sensors (ATIS) (Posch et al., 2011) represent a scene using spatiotemporal data in the form of spikes or motion events at a temporal resolution on the order of microseconds. These spikes are created as a result of objects moving in the scene, camera ego motion or both. Due to the high dynamic range and asynchronous data capture of this class of sensors, problems of illumination and frame-rate are inherently solved before data processing.

Several methods have been proposed to address tracking of moving objects using neuromorphic vision sensors. Litzenberger et al. (2006) used a mean shift approach to identify and update clusters or blobs of events generated by moving vehicles. Piatkowska et al. (2012) used a Gaussian mixture model (GMM) to locate and update clusters of people moving in a scene. Ni et al. (2012) presented an interesting application where motion was segmented to generate haptic feedback for a micro-gripper setup. Their method used a nearest point matching strategy to update the position of a predefined template for a gripper and circle object to be tracked. A DVS based particle tracking algorithm was demonstrated by Drazen et al. (2011) using a spatiotemporal window. Valeiras et al. (2015) showed real-time face tracking using neuromorphic imagers. Their method used spring-like interactions between Gaussian trackers to maintain uniformity between various feature points being tracked. However, the algorithm requires a predefined model of the object. Lagorce et al. (2015) used a multi-kernel based approach to perform invariant real-time multi object tracking. A combination of kernels were defined and used to describe object parts (e.g., edges) to be tracked. The motion segmentation problem has also been formulated as one of determining salient regions in spatiotemporal data. Rea et al. (2013) implemented a selective saliency model on the iCub platform (Metta et al., 2008), using multiple bottom-up feature maps responsible for contrast, orientation and motion. Serrano-Gotarredona et al. (2009) illustrated a parallel very large scale integrated (VLSI) system using the address-event representation (AER) framework, called convolution AER vision architecture for real-time systems (CAVIAR), for object recognition and tracking. Such event based clustering and spiking neural network (SNN) approaches solve the problem efficiently. However, in these segmentation and tracking applications the sensor was fixed with respect to the moving foreground objects, making segmentation tractable for a small class of implementations. Reverter Valeiras et al. (2015) describe a neuromorphic 3D pose estimation algorithm applicable to tracking moving objects. Although this algorithm is robust in the presence of sensor ego-motion, it needs a pre-defined object model and an estimate of its initial pose. Giulioni et al. (2016) developed a SNN approach utilizing precise spike times to estimate motion. Their method uses an analog chip to estimate optical flow for computation of motion amplitude and direction in real-time. However, the system also assumes no camera motion and, therefore, it is not suitable for applications where camera motion is involved.

The motion segmentation methodology proposed in this paper utilizes small controlled movements of a sensor to enable synchronization of spike events, which permits discrimination of spatiotemporal motion events as static background or dynamic foreground. The human somatosensory system was the inspiration for this approach, where micro-movements precede and prepare acquisition of signals through sensory organs such as the eyes or ears. Saccades are characterized by small rapid eye movements to focus on the object of interest. It is well-known that mammals use saccades for vision and insects use ear motion for source localization (Miles et al., 1995). Saccades are an important part of visual processing, however, it is unclear if they facilitate motion segmentation (Ahissar and Arieli, 2001; Martinez-Conde et al., 2004; Rolfs, 2009). Miles et al. (1995) found experimentally how mechanical vibrations of interaural tympana induce a temporal difference in closely spaced sound waves to facilitate source localization with high precision. The application of micro-motor movements for visual sensory perception has been described in the literature as the resonant retina (RR) (Hongler et al., 2003) and dynamic retina (DR) (Prokopowicz and Cooper, 1993). Hongler et al. (2003) demonstrated that micro-saccades are useful for edge detection and object segmentation tasks, which is biologically similar to the phenomenon of stochastic resonance. Prokopowicz and Cooper (1993) proposed DR as a technique that employs perturbations of mobile robots to enhance spatial data processing. Image sensors perturbed by vibrations or noise have been used for spatial enhancement through edge, luminance and contrast detection (Landolt et al., 2001; Greschner et al., 2002; Hennig et al., 2002; Donner and Hemilä, 2007; Rucci et al., 2007; Yi et al., 2011).

We contribute to this body of work by developing a novel methodology to enable motion segmentation using a neuromorphic vision sensor. In typical applications, a moving sensor will induce events or spikes from an entire scene, making segmentation a challenging problem. These spikes are an artifact of self-motion. When a sensor is placed on a moving platform, both moving (dynamic) foreground and (static) background objects generate spikes. In this paper, we address the problem of classifying spikes or motion events into foreground and background events by taking advantage of an induced sensor micromotion. To compute reliable temporal statistics that are a consequence of micromotions, we introduce the concept of *spike-groups*. They are clusters of spikes that appear in predefined space-time voxels. These elements help capture relevant scene statistics as a result of micromotions and are central to our classification strategy. We show that a micromotion sensor strategy removes spatial uncertainty by taking advantage of the high temporal resolution of neuromorphic imagers.

The contributions of this paper are as follows. (1) Utilization of induced micromotion movements to facilitate efficient sensory visual perception. (2) Development of a novel asynchronous signal processing technique that exploits temporal statistics. To the best of our knowledge, this is the first work that presents an active learning framework for motion segmentation that addresses foreground-background separation using an event based sensor. Specifically, the algorithm uses motion events as inputs and assigns a binary label indicating its category (background or foreground) as the output. Our method for motion segmentation was implemented on a wheeled robot and the results demonstrate that it is capable of real-time segregation of objects from background under arbitrary camera motion.

The organization of this paper is as follows. Details of our saccade based approach to classify dynamic spatiotemporal data and the concept of spike-groups are described in Section 2. In Section 3 we give details about the controlled experiments performed to quantify our algorithm's performance and provide a discussion about our findings. Section 4 provides a deliberation about the implications of the concepts introduced in this study and their future applications.

## 2. Materials and methods

**Figure 1:**
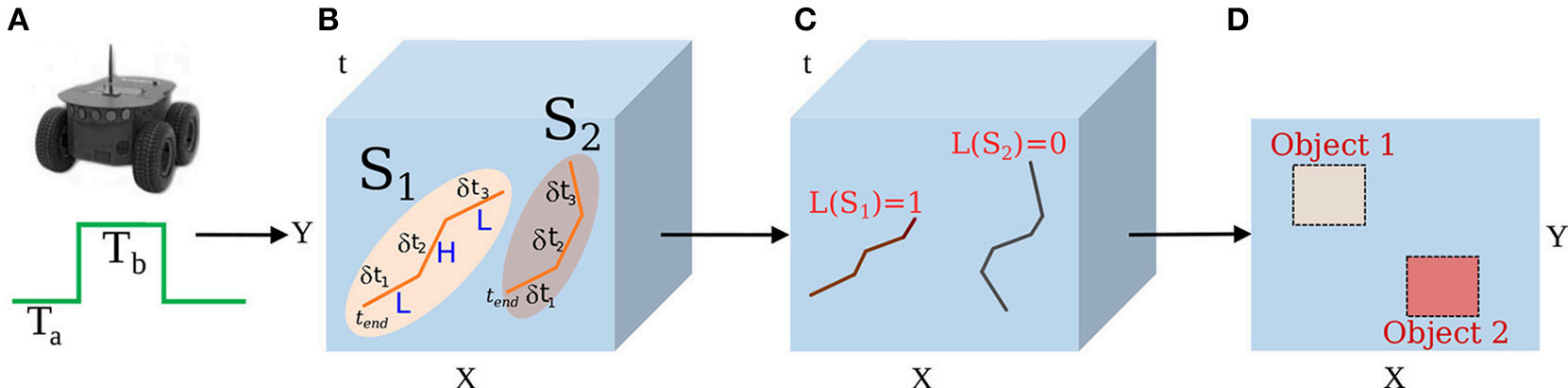
**Motion segmentation procedure using a saccadic motion profile with a neuromorphic imager. (A)** A camera is mounted on the platform to follow a velocity profile (square pulse). **(B)** The motion generates spikes at pixels locations of the vision sensor. Spikes that are spatiotemporally close typically belong to the edge of a single object. Spike-groups are formed from such neighboring pixels. **(C)** Spike-groups are annotated to belong to a background or moving object category. **(D)** Labeling spike-groups in space-time allows for their classification as 2D representations.

In this section, we provide a block diagram of the overall system and mathematical foundation for spike-groups and assignment functions. In addition, the utility of motion segmentation to classify elementary components of micromotion employing temporal statistics is discussed. Figure 1 depicts a vision sensor mounted on a wheeled platform while performing segmentation. A novel feature of our system is that the speed of the robot is jittered by periodic square velocity pulses (or micromotions). This velocity profile has two levels or phases; a normal and low velocity phase. During a micromotion cycle, the robot remains in low velocity phase for time interval *T_a_* μs, and normal velocity phase for *T_b_* μs. Data analysis is performed after each micromotion cycle or at specific time intervals to construct spike-groups as discussed below.

### 2.1. Event based vision sensors

Event based vision sensors such as the DVS and ATIS emulate biological principles on a silicon chip. The DVS is an array of 128 × 128 pixels with a maximum response time of 1μ s. Each pixel responds dynamically and independently to log intensity changes in a scene. This provides asynchronous spatiotemporal data of motion events denoted as

(1) $${𝕄}_{i}={\left(x,y,\phi ,t\right)}_{i}\text{ for }i=1,2,\ldots ,n,$$

where *x, y* are the “spiking” pixel location and ϕ denotes an intensity change at time *t*. The data obtained from a sensor is represented as

(2) $$D=({𝕄}_{1},{𝕄}_{2},\ldots {𝕄}_{i}),$$

where *D* is the spatiotemporal data represented as a set of motion events with increasing timestamp values. Due to the precise temporal and asynchronous nature of neuromorphic vision sensors, it is necessary to develop a mathematical framework to analyze such data without loss of information.

### 2.2. Spike-groups: definition and properties

**Figure 2:**
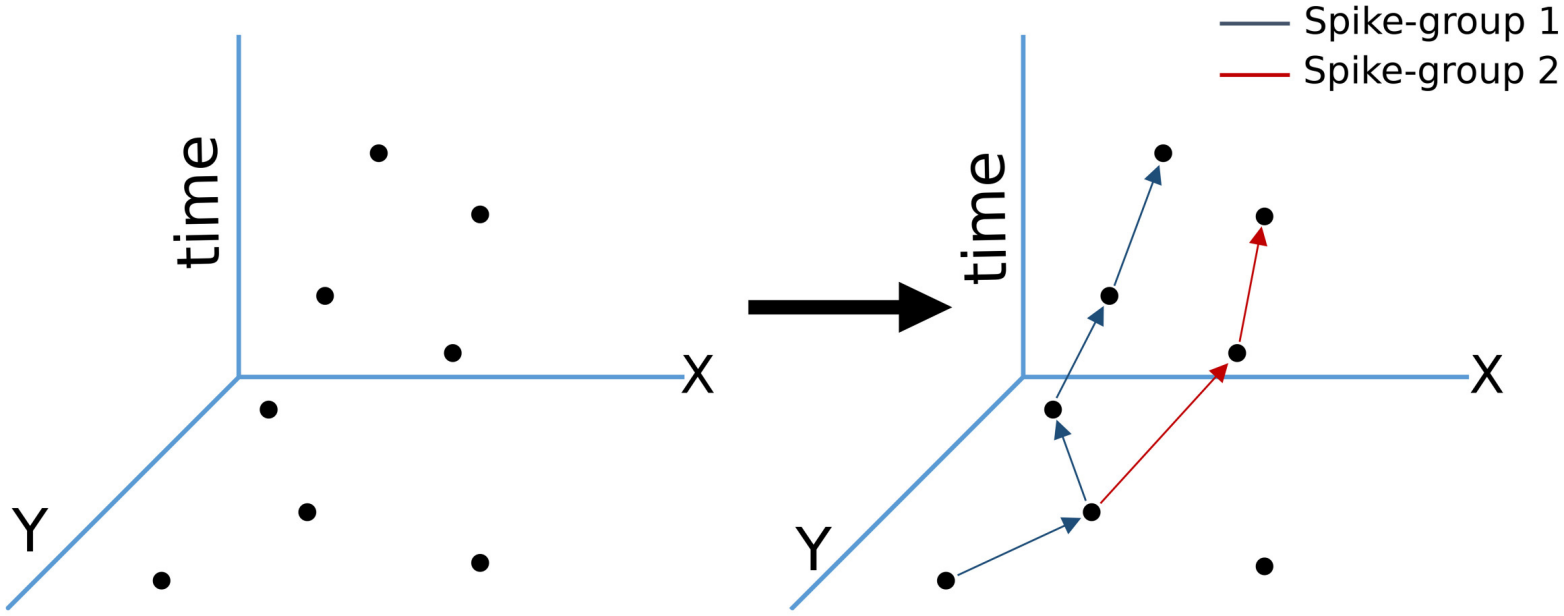
**Spike-groups based spatiotemporal data clustering**. Spike-groups are redundant clusters formed from motion events as they occur. The particular spike group assignment criteria results in a diverging tree like structure (more than one pixels can share nearby statistics). Arrows represent increasing time.

Our motion segmentation technique exploits small controlled square pulses perturbing the robot's motion to differentiate static and dynamic information. The premise of micromotion is based on the observation that when a camera is in motion, events due to the static background are correlated with self-motion. However, motion events associated with moving objects are not consistent with a camera's movement profile. This is because dynamic objects have relative velocities with respect to the camera, but un-correlated to the camera's motion profile. This feature, coupled with the temporal accuracy and precision of neuromorphic sensors, enables the estimation of temporal correlations to separate such movements types. For statistics estimated using spatiotemporal data to be useful in practical applications, they must be updated in near real-time when new motion events arrive from a sensor. For this reason, we develop the concept of spike-groups, which are a factorization of the spatiotemporal data, *D*, into meaningful motion components. Figure 2 illustrates that spike-groups are close spatio-temporal clusters of constant cardinality motion events delivered by the sensor. The intrinsic aggregation rule underlying this definition is computed using an assignment function based on statistics of successive temporal spike differences at neighboring pixel locations. Spike-groups are represented as

(3) $$S=(\delta t_{1},\delta t_{2},\ldots ,\delta t_{q}),$$

where *S* ∈ ℤ^*q*^ denotes the vector containing temporal differences, δ*t*, of motion events. For computational purposes, spike-groups are stored at pixel location where the latest motion event occurred. As a consequence of this definition of spike-groups, the temporal differences in Equation (3) do not necessarily belong to only one pixel location, instead they span various neighborhoods in space-time, which we denote as space-time voxels. The spatiotemporal motion event data, *D*, can also be written as

(4) $$D=\underset{j}{\cup }S_{j}\text{ or }D=(S_{1},S_{2},\ldots ,S_{k})\text{ where }j=1,2,3,\ldots ,k$$

and *k* is the number of spike-groups in the data. The advantage of spike-groups is that the incoming sensor data is divided into locally meaningful groups, which is important to identify objects in the scene. Due to the high temporal resolution of these sensors, spikes that are spatiotemporally close may represent the same shape and likely the object edges. Hence, every object can be described by a combination of spike-groups, simplifying the task of motion segmentation. This also simplifies object segregation to one of spike-group formation from motion events by formulating appropriate discriminant local statistics. These statistics allow for characterization of a spike-group as belonging to one of two classes (moving object or background) in our application but may be generalizable to higher orders.

Some of the important characteristics of spike-groups are summarized below.

1. Spike-groups are fixed length vectors composed of time differences of motion events.
2. A motion event belongs to at most one spike-group but the converse is not true, i.e., a spike-group can be assigned to more than one motion event, if the assignment criteria is satisfied. Hence, spike-groups are non-disjoint sets, where a spike can contribute to the statistics of more than one spike-group.
3. For each motion event, the optimal (or best suited at the time) spike-group is assigned to it. The spike-group is then copied to the new motion event's pixel location. This is followed by shifting spike-group components to account for new temporal difference elements.

### 2.3. Spike-groups: assignment

**Figure 3:**
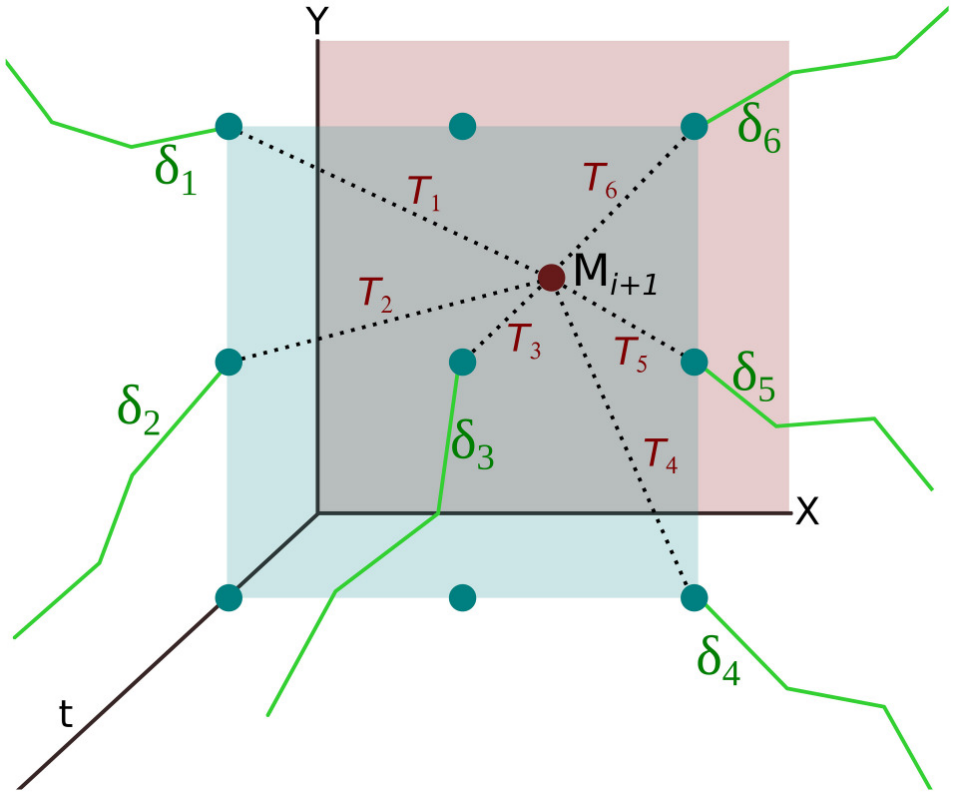
**Formation of spike-groups using hypothesis based spatio-temporal clustering**. For every new motion event 𝕄_*i*+1_, each spike-group (solid green lines) in its neighborhood generate a hypothesis *T*_*i*_ about its inclusion (dotted black lines), if they lie within the temporal window of the event parameter ρ. The hypothesis pertains to the difference or log ratio between the temporal difference of the last element with the current motion event (*t*_*i*+1_−*t*_*last*_) and last two motion events comprising the spike-group (δ*t*_*q*_). The hypothesis with a minimum value is selected for inclusion in this motion event.

When a new motion event, 𝕄_*i*+1_, occurs its profile is compared with spike-groups at all pixel locations in its spatiotemporal neighborhood, N. This neighborhood is defined as a *n* × *n* square matrix. The motion event is assigned to an existing spike-group $S_{j}$ in the neighborhood if (1) the temporal difference is the same as the last two motion events and (2) it is smaller than a threshold ρ. This assignment is formalized as a inclusion test, *T*(·), that is, the motion event 𝕄_*i*+1_ is assigned to the *j*th spike-group $S_{j}$ if

(5) $$T({𝕄}_{i\;+\;1}\in S_{j})=\left|(t_{i\;+\;1}-t_{last}^{j})-\delta t_{q}^{j}\right|\text{ if }t_{i\;+\;1}-t_{last}^{j}\leq \rho ,$$

where |·| is the absolute difference operation, *t*_*i*+1_ is the current motion event's occurrence time, $t_{last}^{j}$ is the occurrence time of last event of the spike-group, $\delta t_{q}^{j}$ is the last element of spike-group $S_{j}$ and ρ is the temporal size of the neighborhood N. Figure 3 illustrates that for each motion event, the assignment function *T*(·) (i.e., Equation 5) is evaluated at each pixel in its spatial neighborhood. The event belongs to spike-group $S_{j}$ if

(6) $$j=\arg\text{​ }\underset{\forall S_{j}\in N}{\min}T({𝕄}_{i\;+\;1}\in S_{j})$$

where *j* ∈ [1, *n*^2^] is the index of neighborhood pixel location to which the new event is assigned. If none of the hypotheses in the neighborhood are found to be true, a new spike-group is started at the pixel location of spike 𝕄_*i*+1_, with that motion event being its first element. The temporal difference operation (see Equation 5) is simple to estimate in real time and facilitates proper class characterization, however, is not robust to noise. From experimental observations, a better hypothesis for spike-group assignment is

(7) $$T({𝕄}_{i\;+\;1}\in S_{j})=\left|\log\frac{(t_{i\;+\;1}-t_{last}^{j})+\lambda }{\delta t_{q}^{j}+\lambda }\right|\text{ if }t_{i\;+\;1}-t_{last}^{j}\leq \rho ,$$

where λ is a regularization parameter. Spike-groups are classified only when *q* events have occurred to reflect statistics from both velocity phases of the saccadic motion profile. A resulting effect of spike-group formation is that spatiotemporal data are arranged into maximally correlated components with respect to their velocity profile, which is a critical step for classification of labeled groups. Assignment of motion event 𝕄_*i*+1_ to a spike-group involves copying the spike-group at the minimum hypothesis position, *j* (see Equation 6), to this new motion event's position (*x*_*i*+1_, *y*_*i*+1_). An alternative interpretation of this strategy, useful for the next step, is to assume that all spike-groups are hypothesis sets that compete for assignment of the most recent spike event (Figure 3).

### 2.4. Spike-groups: classification strategies

After motion events are assigned to spike-groups, the goal is to assign class labels as background or moving object. Due to internal program setting (discrimination between two classes), an expeditious methodology is to build a non-parametric classification metric $F\left(S_{j}\right)$ for the spike-groups and apply traditional pattern classifiers by modeling the distribution of F(·) as a mixture with two components. Spike-groups belonging to a moving object class have temporal differences that are influenced primarily by its own motion, with some contribution from the micromotion profile. However, spike-groups belonging to the static background class have temporal differences that are completely caused by the robot's motion profile. Therefore we anticipate that there is a bimodal distribution over the space of spike-group measurements.

In this paper, we describe two methods to discriminate between background and moving objects using either a parametric or non-parametric approach applied to spike-groups. Both methods use non-labeled data to perform clustering in an unsupervised manner. The only information imposed in the solution is the existence of two classes (background and moving objects), which implies modeling the measurements as a mixture of two clusters, with each cluster being updated in time interval batches of 1 s. For a proof-of-concept, we select data that have only one moving object in the scene leading to the assumption of a bimodal distribution over F(·). We select two different approaches to define the metric F(·) for discrimination of the measurements; (1) standard deviation and (2) Renyi's entropy. Therefore, for each new spike-group vector the assignment is reduced to a single scalar decision, either minimization of standard deviation or entropy.

#### 2.4.1. Discrimination using standard-deviation

The standard deviation of a spike-group is defined as

(8) $$F(S_{j})=\sqrt{\frac{\sum_{\forall i}{\left(S_{j}(i)-\mu_{j}\right)}^{2}}{q-1}},$$

where $S_{j}\left(i\right)$ is the *i*th element in the spike-group vector, μ_*j*_ is the mean of time differences within a spike-group $S_{j}$ ($\mu_{j}=𝔼\left[S_{j}\right]$) and 𝔼[·] is the expected value operator. Using standard deviation to characterize spike-groups implies that we are modeling each spike-group measurement as a Gaussian function and their distribution as a Gaussian mixture.

**Figure 4:**
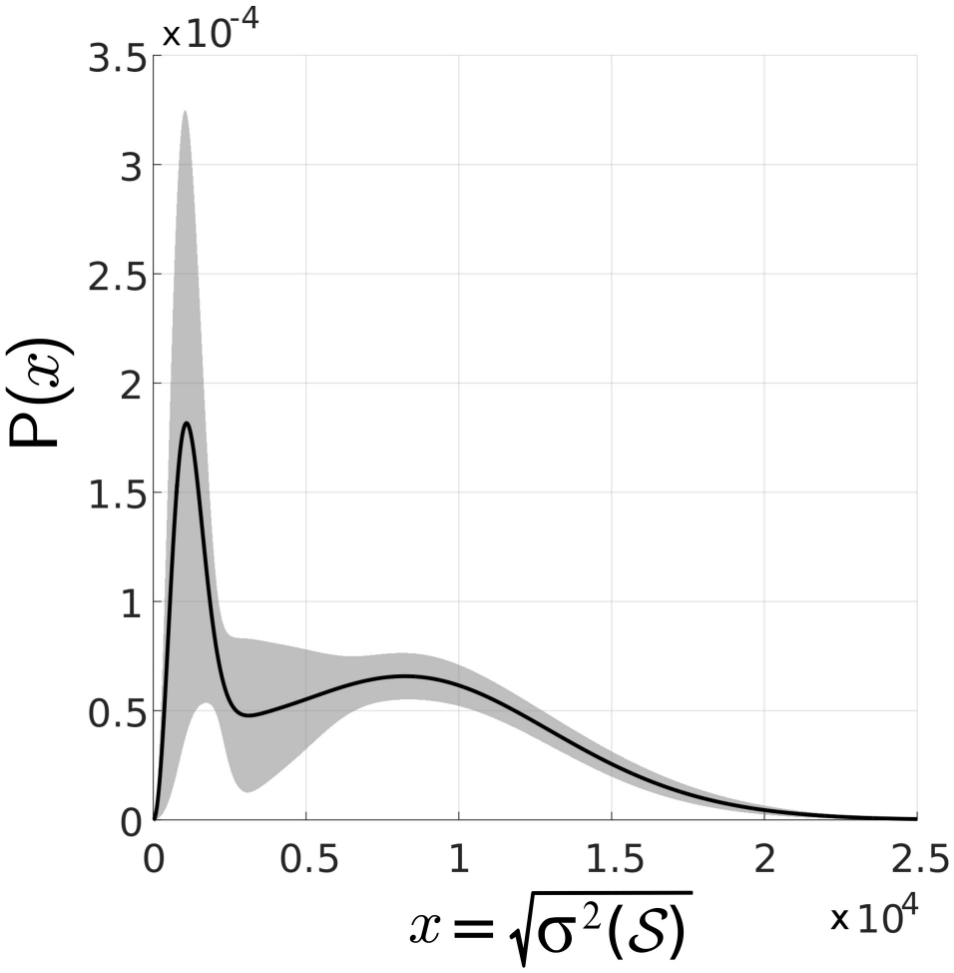
**Distribution of standard deviation of spike-groups**. Empirical distribution of standard deviation is shown as the solid black line and the shaded region is one standard deviation about it. The probability density functions (pdfs) of data across time intervals was captured, followed by calculation of mean at each point on the *x*-axis and one standard deviation around it.

A sample distribution of the standard deviation over a real set of measurements is shown in Figure 4, which illustrates the bimodality of spike-groups formed. The data corresponding to this plot was collected from an oscillating object in an office background with the robot moving toward it. Standard deviation of spike-groups for 1 s non-overlapping intervals was plotted followed by calculation of the mean curve and standard deviation around it.

Since Figure 4 shows that each class has an asymmetric distribution, the Gaussian mixture assumption can be improved. Therefore, the standard deviation of all spike-groups is modeled as the combination of two Maxwell Boltzmann probability distributions as

(9) $$P(F(S_{j})=x)=\beta g(x;\theta_{1})+(1-\beta )g(x,\theta_{2}),$$

where *g* represents a Maxwell-Boltzmann distribution function

(10) $$g(x;\theta )=\sqrt{\frac{2}{\pi }}\frac{x^{2}e^{-x^{2}/(2\theta^{2})}}{\theta^{3}},$$

parametrized by θ_1_ or θ_2_ for the classes foreground and background, respectively, and β ∈ [0, 1] is the class prior. For this choice of mixture distribution, the log likelihood function is written as

(11) $$\log L=\sum_{i\;=\;1}^{N}\sum_{j\;=\;1}^{2}z_{i}^{j}P(w_{j})\left(2\log x_{i}-\frac{x_{i}^{2}}{2\theta_{j}^{2}}-3\log\theta_{j}+C\right),$$

where *C* is a constant, *N* is the number of datapoints (spike-groups), β = *P*(*w*_1_), 1 − β = *P*(*w*_2_), and $z_{i}^{j}$ is a binary latent parameter defined as

(12) $$z_{i}^{j\;=\;1}=\frac{g(x;\theta_{1})}{g(x;\theta_{1})+g(x;\theta_{2})}.$$

By differentiating the log-likelihood function described in Equation (11), the parameters for each class are found as

(13) $$P(w_{1})=\beta =\frac{\sum_{\forall i}z_{i}^{j\;=\;1}}{\sum_{\forall i}z_{i}^{j\;=\;1}+\sum_{\forall i}z_{i}^{j\;=\;2}},$$

(14) $$\theta_{j}=\sqrt{\frac{\sum_{\forall i}x_{i}^{2}z_{i}^{j}}{3\sum_{\forall i}z_{i}^{j}}}.$$

We now describe the algorithm used to incrementally update the parameters as a procedure that is applicable to any robotic setup. For algorithm initialization, data is collected for at least a 4 s duration with a scene comprising of one moving object. This data is used to compute spike-groups followed by an initial classification threshold $C_{init}$ estimate as

(15) $$C_{init}=\frac{\sum_{\forall j}F(S_{j})}{N},$$

where *N* is the number of spike-groups formed from initial data and F(·) is calculated from Equation (8). With this threshold, initial estimates of latent variable $z_{i}^{j}$ for each of the two classes is formulated as

(16) $$z_{i,init}^{j\;=\;1}=\{\begin{array}{ll} 1 & \text{if }x_{i}\leq C_{init},\\ 0 & \text{if }x_{i}>C_{init}. \end{array}$$

Similarly, initial estimate $z_{i,init}^{j=2}$ for the second class is the inverse of Equation (16). Through this definition of the latent variable, initial class parameters as described in Equations (13) and (14) are evaluated. Finally, an update of the parameters is performed through iterative application of Equations (12–14).

Following parameter estimation of the mixture distribution, classification is performed by assigning the new spike-group to the cluster with maximum posterior. This method for parameter estimation and update is based on spatial and velocity constraints assuming that (1) the moving objects cannot disappear suddenly from the scene and (2) the navigation algorithm of the robot implements small gradual velocity changes. In the next section, we describe a method utilizing Renyi's second order entropy to classify spike-groups.

#### 2.4.2. Discrimination using non-parametric Renyi's entropy

Instead of assigning a priori distribution to each one of the class measurements, we use a non-parametric approach. This allows us to use the data samples to estimate the empirical distribution and extract a meaningful feature for discrimination, such as minimization of entropy of the overall measurements. Minimum entropy means that the distribution is a delta function, i.e., all samples clustered at the same point. In our case we assign samples to one of the modes of the mixture distribution such that the entropy of the mixture is minimized. We follow the ideas expressed in Gokcay and Principe (2002) and adapt the algorithm for the simple case of a bimodal distribution, which is simple to implement. Renyi's second order entropy is used as the classification metric for spike-groups in this section. The Renyi's entropy measure for discrete variables is given as

(17) $$H_{\alpha }(P_{S})=\frac{1}{1-\alpha }\log_{2}\left(\int p^{\alpha }(x)dx\right),$$

where *p*(*x*) denotes the probability mass function of the temporal difference values of the spike-group S and *H*_α_(·) denotes Renyi's entropy of order α. Here, we use α = 2 to simplify the estimation (Principe et al., 2000). To estimate *p*(*x*), we apply the kernel density estimator (KDE) with a Gaussian kernel as

(18) $$p(x)=\frac{1}{q}\sum_{k\;=\;1}^{q}\frac{1}{\sigma \sqrt{2\pi }}e^{-\frac{{\left(x\;-\;x_{k}\right)}^{2}}{2\sigma^{2}}}.$$

In Equation (18), the kernel is chosen as a Gaussian parametrized by standard deviation, σ. With this formalization of the kernel, the second order Renyi's entropy, *H*_2_(*P*_S_), is written as

(19) $$H_{2}(P_{S})=-\log\left(\frac{1}{q^{2}}\sum_{i\;=\;1}^{q}\sum_{k\;=\;1}^{q}\frac{1}{\sigma \sqrt{2\pi }}e^{-\frac{{\left(x_{i}\;-\;x_{k}\right)}^{2}}{2\sigma^{2}}}\right).$$

**Figure 5:**
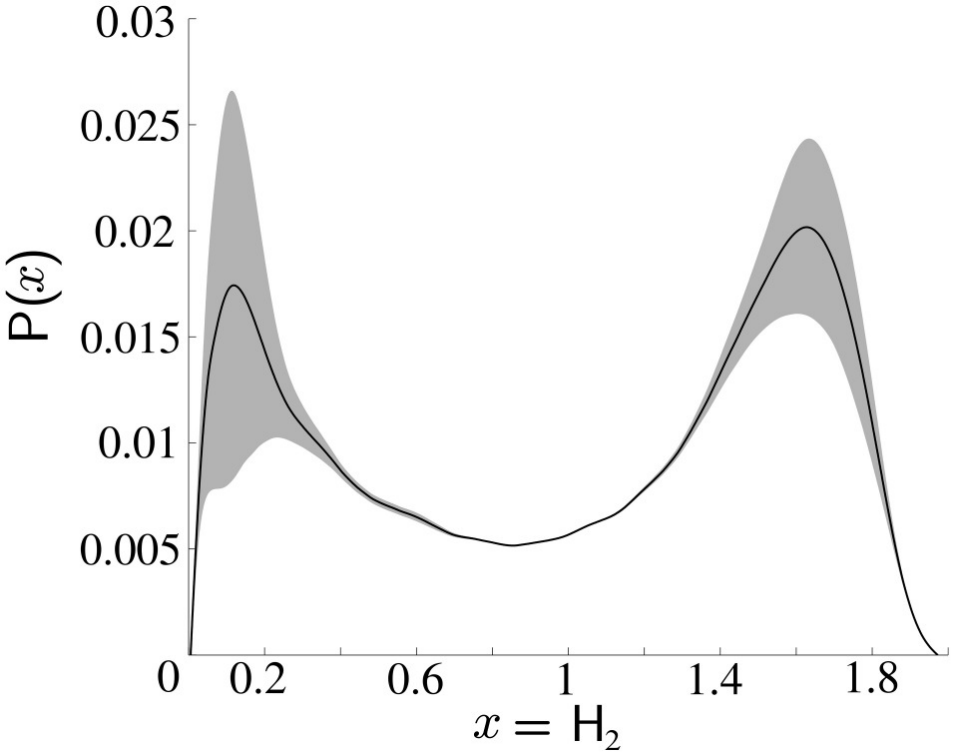
**Distribution of second order Renyi's entropy of spike-groups with a Gaussian kernel**. Shaded region denotes one standard deviation from the mean.

**Figure 6:**
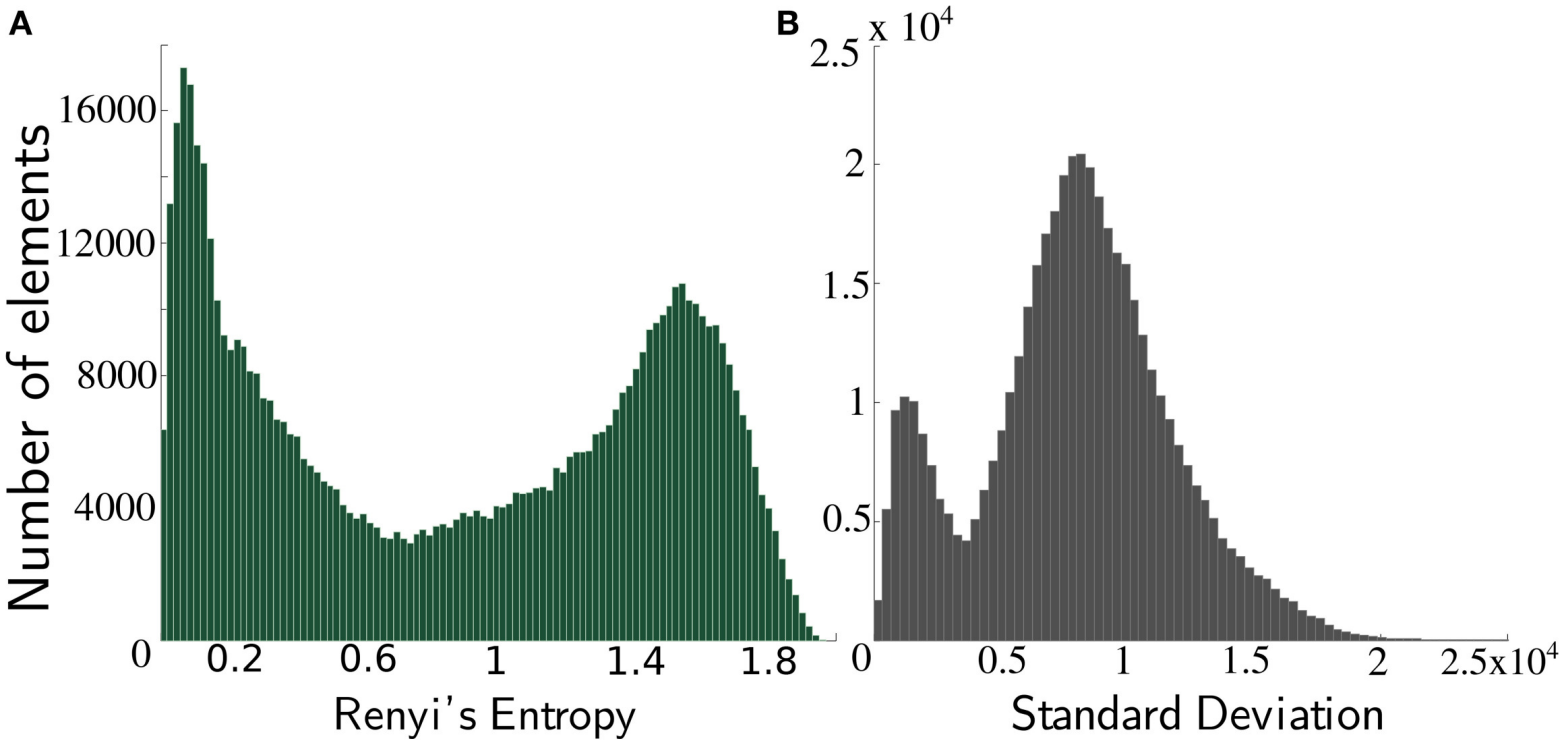
**Sample histograms of Renyi's entropy and standard deviation on spike-groups**. The data comprised an oscillating foreground object in a densely cluttered background. **(A)** The histogram of Renyi's second order entropy is shown. Spike-groups pertaining to foreground class have greater entropy. **(B)** Histogram of standard deviation also shows a clear bimodality. Spike-groups pertaining to the foreground class exhibit lesser standard deviations as indicated by the first peak.

The distribution of *H*_2_(*P*_S_) for spike-groups across a sample data (sets of entropy over 1 s non-over lapping intervals) is shown in Figure 5 and sample histograms are illustrated in Figure 6. A moving object class shows higher entropy values than a background class. The kernel standard deviation σ is an important parameter, which affects the resulting density and entropy calculation. Small values result in an irregular density estimate (over-fitting) while large values do not capture information content properly and result in under-fitting. Initially, data is collected for at least a 4 s duration with the moving object. Spike-groups are calculated and Renyi's entropy evaluated with the kernel parameter setting through the estimator given by Silverman (1986) as

(20) $$\sigma =\frac{1.06\;\min(\sigma_{\delta t},IQR/1.34)\;\eta^{-1/5}}{\sqrt{2}},$$

where σ_δ*t*_ is the standard deviation of the data (δ*t* of all spike-groups), η is the number of data points and *IQR* is the interquartile range of data (Wilcox, 2012). To decide the clusters, a simple test of incremental entropies is sufficient to provide the cluster assignment. With the new spike-group, we compute the change in entropy of each cluster with it. The cluster that displays the least change in entropy is the one that should receive the new spike group because it is the one closer to the mode of the cluster. This cluster entropy is also updated and the process repeats with the new measurement.

## 3. Results

In this section, we present some characteristic results to show the importance of our algorithm and its accuracy for classification.

### 3.1. Datasets created

The following datasets were created and ground truth frames labeled to quantify algorithm performance and find optimal parameter values. The first dataset was used to understand the dependency of the algorithm on the robot's velocity while the second dataset was used to evaluate the importance of micromotion control parameters.

**Figure 7:**
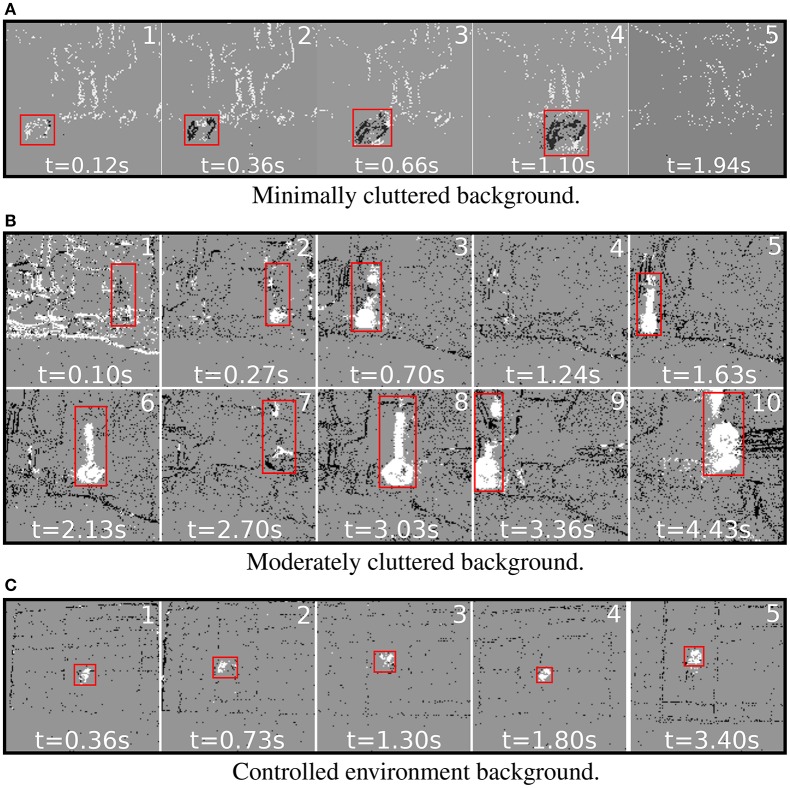
**Labeled frames after segmentation for experiments performed with varying backgrounds**. Red bounding boxes represent the ground truth. **(A)** A sliding box was used as the moving object for the minimal background clutter case. The black pixels are labeled as moving object category. **(B)** Sample frames from dataset-1 post classification. The white regions are spike-groups with a class label as moving object and black regions have a class label as static background. **(C)** Sample frames from dataset-2 post classification. White represents moving object category labels.

1. **Dataset-1**: Data from an oscillating pendulum object was recorded at varying velocity transition levels of 60–80, 60–90, 60–100, 70–80, 70–90, and 70–100 rotations per minute (rpm). The wheel diameter of the robotic platform was 5 cm. The data were collected at *r* = 0.5 and *f* = 10 Hz (see Equation 23). This dataset was recorded with both high and low background clutter (see Figure 7). Using an oscillating object in the dataset allowed for a wide variety of object velocities, from 0 to a maximum pendulum velocity of 1.28 m/s. The pendulum length was 1.4 m and the initial release angle was π/9 radians.
2. **Dataset-2**: To quantify the effect of micromotion parameters on classification accuracy, we collected data in a controlled environment. The background was made up of black stripes (10 cm each) on a white board, with a horizontal and vertical separation of 15 and 10.5 cm, respectively. The foreground object was a ball with a 5 cm diameter rotating on a 6 cm rod. During experiments the frequency of micromotions was decreased with various rotational speeds of the foreground object, from 10 to 30 cm/s in increments of 5 cm/s.

To evaluate our classification technique's performance using standard computer vision methods, the datasets were binned at 30 fps and the ground truth labeled as rectangular regions about the moving object in the scene.

### 3.2. Accuracy of segmentation

Sample segmentation results using our motion segmentation algorithm on various types of recorded data are shown in Figure 7 and Supplementary Video. For each experiment, the corresponding ground truth label is denoted by a red bounding box. Figure 7A shows sample segmentation results on an object moving straight across the sensor's field of view (FoV) at a speed of 5 cm/s. Figures 7B,C show sample segmentation results on experiments from dataset-1 and dataset-2, respectively. Below we summarize the salient points of this methodology by visually analyzing the segmentation produced.

1. Frame 1 in Figures 7A,B denote the initialization phase of our algorithm. During initialization all motion events are labeled as background category. When motion events are detected spike-groups are formed, leading to rapid class distinction.
2. Our technique performs dense labeling of the exact object shape instead of calculating a bounding box. In computer vision the process of finding probable object regions is termed as region proposal and is a computationally expensive process (Girshick et al., 2014; Girshick, 2015; Ren et al., 2015). Our proposed motion segmentation methods can enable neuromorphic systems to perform complex tasks such as object segmentation and recognition in near real-time.
3. In Figure 7B frames 2 and 7 show that when the oscillating object reached 0, velocity segmentation accuracy decreased.
4. Since Figure 7A, frame 5 and Figure 7B, frame 4 have no moving objects within the sensor's FoV, the number of false positives is low.

**Figure 8:**
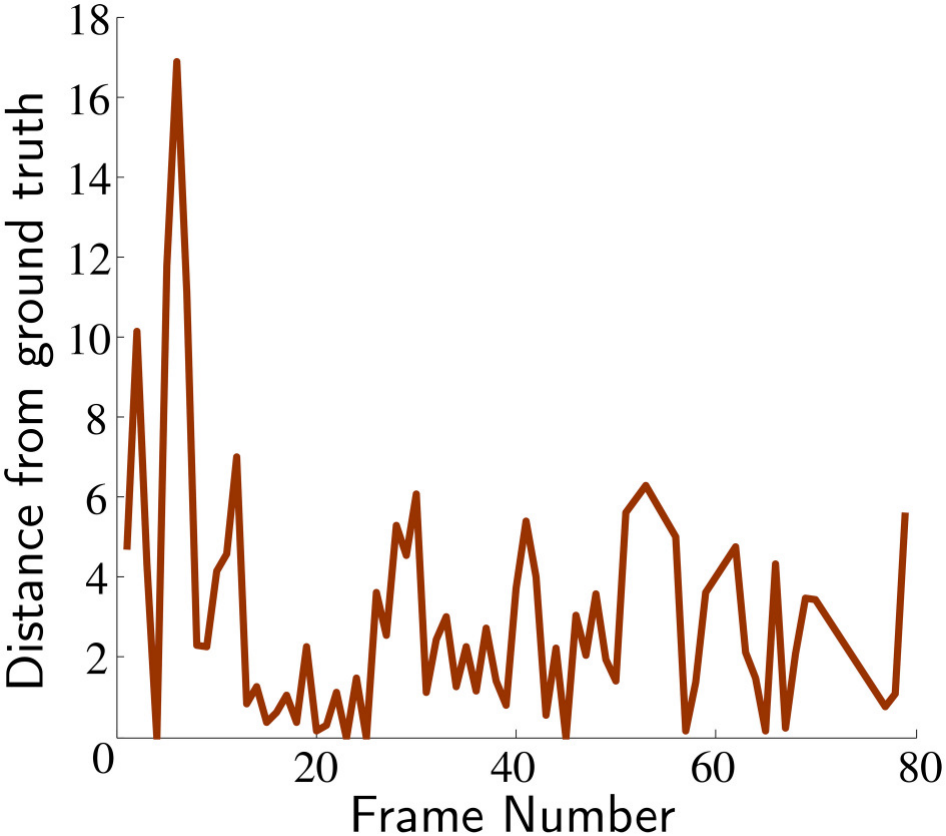
**Deviation of segmentation center of mass from true object center**. The classified center of mass was within a 8 pixel distance at all times across the dataset-1 (except algorithm initialization).

Figure 8 provides a precision plot (Babenko et al., 2011; Wu et al., 2013) that illustrates deviations by our algorithm from the ground truth center of mass (using the standard deviation method). The Euclidean norm is used as the metric to assess the algorithm's robustness against misclassification errors. These results demonstrate that the calculated center of mass is always within 8 pixels after the initial error spike (post algorithm initialization), with a mean of 5 pixels and standard deviation of 1.8.

### 3.3. Experiments in a controlled environment

**Figure 9:**
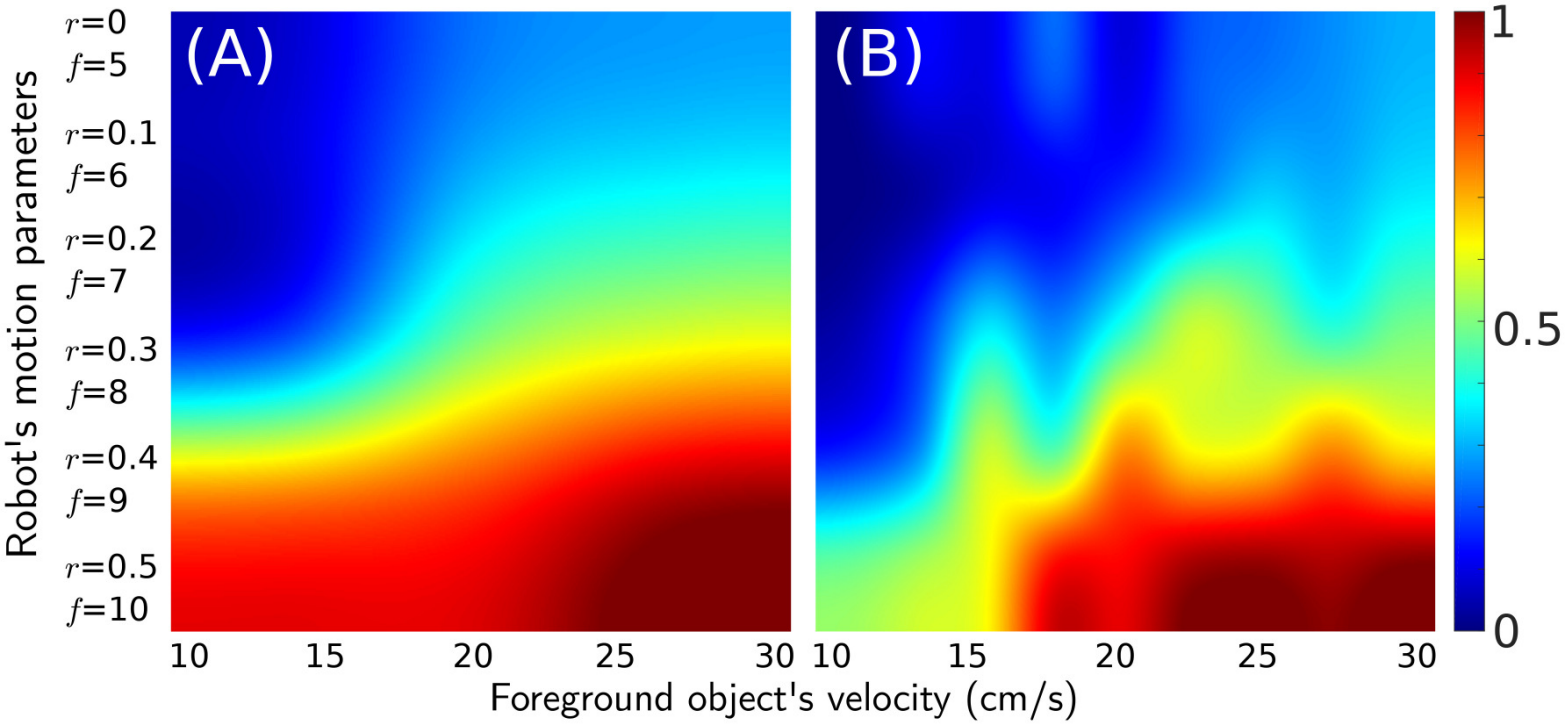
**Performance map for experiments in a controlled environment (dataset-2)**. The vertical axis represents decreasing parameter values for *r* and *f*. The horizontal axis represents rotating objects moving with increasing velocity. The color at any point represents classification performance for a given parameter set using **(A)** second order Renyi's entropy metric and **(B)** standard deviation metric.

For each trial in dataset-2 the robot's movement was dictated by the parameters given in Figure 9. For each parameter setting of the moving object (10–30 cm/s in increments of 5 cm/s), it permits identification of the best solutions across the dataset. The classification process for this dataset was performed using standard deviation and Renyi's entropy methods. For each trial, its performance was evaluated with respect to the labeled ground-truth (fraction of frames for which predicted number of true positives is greater than or equal to 0.8 times the ground truth true positives number). The performance calculation for dataset-2 was followed by interpolation to yield the image in Figure 9. Each point of the image represents an experiment of the dataset with corresponding motor action parameters and moving object velocity. The vertical axis represents parameters *f* and *r*, that correspond to the frequency of micromotions and the time interval between velocity transitions of the robot, respectively. The horizontal axis represents rotational velocity of foreground objects from 10 to 30 cm/s. Along the vertical axis the values of parameters *r* and *f* decrease gradually from *r* = 0.5 and *f* = 10 to *r* = 0 and *f* = 5, which illustrates that moving up the vertical axis the micromotion profile of the robot decreases in each trial. In addition, this plot serves as the ground truth and highlights several important features about the algorithm, which are enumerated as follows.

1. Lower foreground object velocity results in reduced classification accuracy. However, when the foreground object velocity increases, classification accuracy improves. This is because the foreground and background distributions overlap less.
2. Motor control parameters (vertical axis) with higher values (*f* = 10 and *r* = 0.5) yield the best performance for all velocities of foreground object. This illustrates the importance of micromotions for classification. For higher foreground object velocities, the performance deteriorates along the vertical axis. This is because it is necessary for the robot to spend proportional amount of time in high and low velocity phases to accumulate sufficient characteristic temporal differences for spike-groups for each class. For higher object velocities the time required to accumulate discriminative information is lower. See the Supplementary Video for results of object classification at various points on the performance map.
3. Classification with Renyi's entropy metric gives more consistent performance across the foreground object's velocity and extends the range of velocities where performance is good (below 15 cm/s).

Table 1 shows the detection rates for the two methods over recordings in dataset-1. For each recording, data is binned at 30 fps post classification. A frame is counted as positive detection if the number of reported true positives is greater than or equal to 0.8 times the ground truth true positives number. From the table, it is observed that the entropy method performed slightly better than the standard deviation method. This might be a result of the non-parametric density function used in clustering the spike-groups (Gokcay and Principe, 2002) that avoids errors in fitting of the mixture distribution.

**Table 1:** **Detection rates**.

**Robot Velocity Levels (rpm)**	**Renyi's entropy method (%)**	**Standard deviation method (%)**
60–80	86.05	85.01
60–90	87.07	85.05
60–100	90.50	87.40
70–80	86.20	84.40
70–90	91.20	89.80
70–100	92.50	92.00

### 3.4. Evaluation of saccadic motion

**Figure 10:**
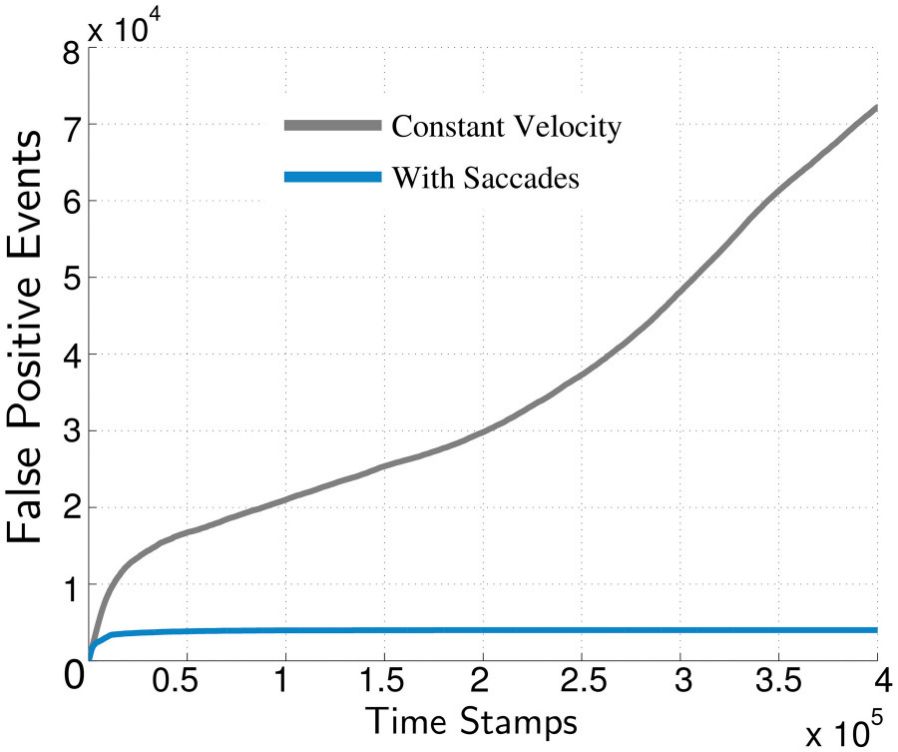
**Importance of micromotions for sensory perception using spike-groups**. Gray line: Cumulative false positives without micromotions. Blue line: Cumulative false positives classified using micromotions.

The saccadic motion profile (jitter) employed is an important prerequisite for the spike-groups to be discriminative. The discriminative power of spike-groups is achieved primarily during velocity transitions of the robot. As the sensor changes velocity levels, the elements of spike-groups associated with background objects will reflect this change in their temporal differences. Since moving objects have a velocity of their own, the spike-groups exhibit a smaller change. This behavior manifests itself as the bimodality observed in the data. To emphasize the importance of micromotions we performed the following experiment. We analyzed a scene having only background and no moving object. In the first case, the data was captured while performing micromotions on the robot. In the second, data was captured without performing micromotions on the robot. We expected in the second case there would be large misclassification errors at all times, since the spike-groups formed would not be informative (discriminative). The results of applying a spike-group based classification approach using Renyi's entropy classification metric is shown in Figure 10. The cumulative number of false positives remained constant when using saccades (initial misclassifications at algorithm initialization) but increases exponentially without saccades.

### 3.5. Free parameters

To achieve robust classification, many free parameters need to be determined before an experiment. In this section, we provide details of each parameter and its effect on the algorithm. Guidelines are given to select optimal parameter values to enhance performance. These values are shown in Table 2 and determined using information from neighboring pixels and estimates from observed data.

**Table 2:** **Optimal algorithm parameter values**.

**Parameter**	**Reference**	**Optimal value**	**Short description**
*q*	Equation (3)	27	Maximum number of elements in each spike-group.
ρ	Equation (5), Equation (7)	5 × 10^4^μ*s*	Temporal window size.
N(n×n)	Equation (6)	*n* = 3	Spatial window size.
λ	Equation (7)	0.1	Regularization factor in log hypothesis.
α	Equation (17)	2	Order of Renyi's entropy used.
*T*_*b*_	Equation (23)	5.0 × 10^4^μs = 0.050s	Normal velocity phase saccade time duration.
*T*_*a*_	Equation (23)	2.5 × 10^4^μs = 0.025s	Low velocity phase saccade time duration.
*r*	Equation (23)	0.5	Ratio of low and normal velocity phase durations.
*f*	Equation (23)	10 Hz	Number of saccades (micromotions) per second.

#### 3.5.1. Spike-group length

**Figure 11:**
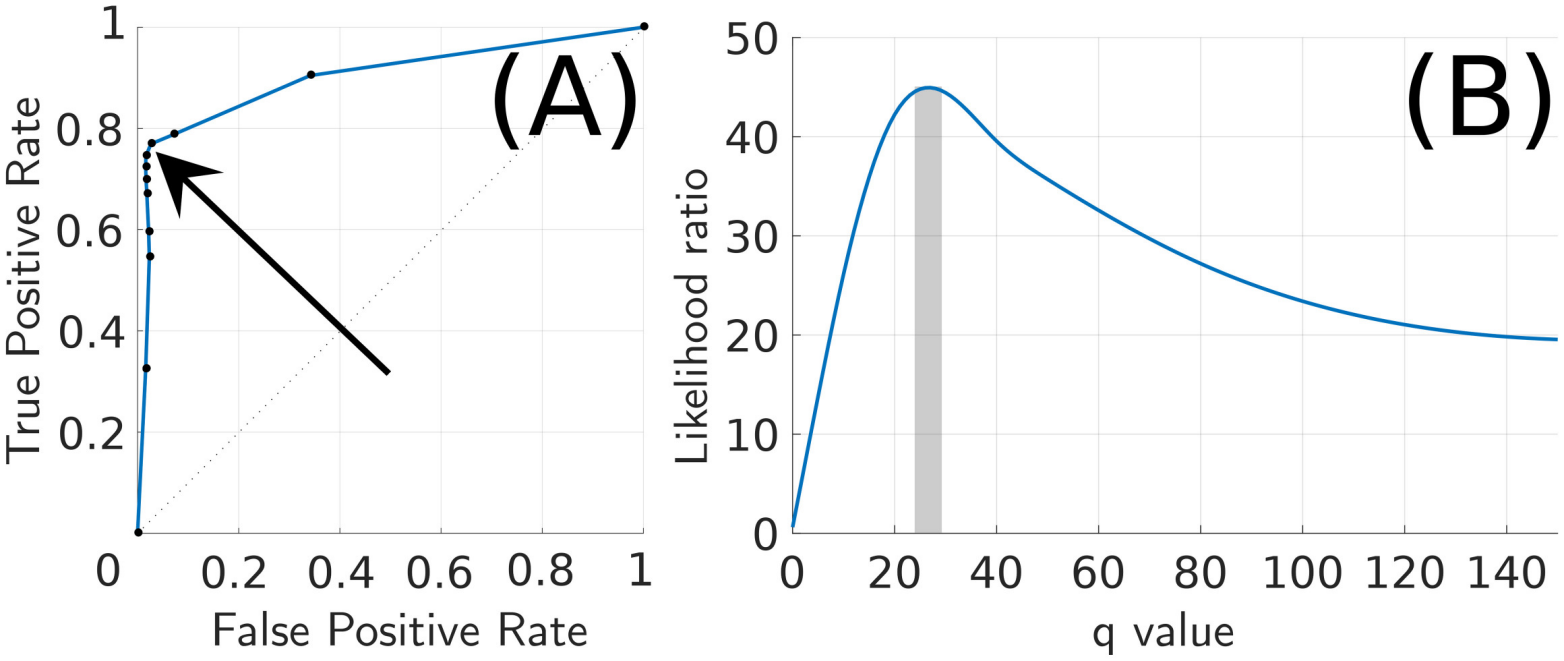
**Likelihood ratio and ROC curve for parameter *q***. **(A)** ROC curve for parameter *q* (arrow indicates *q* = 27). **(B)** Equivalent likelihood ratio curve for parameter *q* has a distinct maxima near 27.

The number of elements in a spike-group is critical for robust classification since it determines the amount of discriminative information as a result of velocity transitions. To estimate the optimal spike-group length a receiver operating characteristic (ROC) (Metz, 1978) curve was computed for dataset-1 (Figure 11A). The corresponding positive likelihood ratio curve is shown in Figure 11B. The positive likelihood ratio (LR) (Choi, 1998) metric is defined as

(21) $$LR=\frac{TPR}{FPR},$$

where TPR and FPR are the true positive rate and the false positive rate, respectively. The operating point was chosen as the maxima of the LR curve around *q* = 27 (solid vertical bar in Figure 11B). The corresponding operating point in the ROC curve is indicated by the arrow in Figure 11A. An example of spike-groups formed from a trial in dataset-1 is shown in the Supplementary Video.

A diverging tree like structure of spike-groups is a result of their particular assignment algorithm, allowing neighboring pixels to share spatio-temporal statistics. Since foreground objects have connected structures (edges) moving coherently, spike-groups sustain for a longer time compared to background objects. In cases of a highly structured background scene such as dataset-2, spike-groups are differentiable through their temporal statistics induced by robot micromotion.

#### 3.5.2. Spatiotemporal parameters

Spatiotemporal parameters play an important role in the assignment of spike-groups. Parameter *n* specifies the spatial length of a window that is square, symmetric (N=n×n) around the current motion event location (Figure 3). Increasing the window size allows for spike-groups from greater spatial distances to be assigned to the current event's location. This is often not desirable since spike-groups represent parts of edges from a foreground or background object. A larger window size allows spatial discontinuities in spike-groups resulting in classification errors. We have used an optimal spatial window size that was 3 × 3 around an event location.

Parameter ρ controls the temporal component of the spatiotemporal window, which can be shown as

(22) $$max_{\forall i}(S_{i})\leq \rho ,$$

where $S_{i}$ represents the *i*th element of spike-group S. This parameter represents the upper limit a spike-group can have. Hence, during hypothesis formation (Equations 5, 7), if none of the hypotheses evaluate to a definite value, the motion event is categorized as noise. In our experiments, we found the optimal temporal window size to be 5 × 10^4^μ*s*, which allowed the non-moving object category spike-groups to be rejected as noise and others classified as background. Due to the filtering effect of this parameter, it was not necessary to use a background activity filter.

#### 3.5.3. Kernel parameter and Renyi's entropy

**Figure 12:**
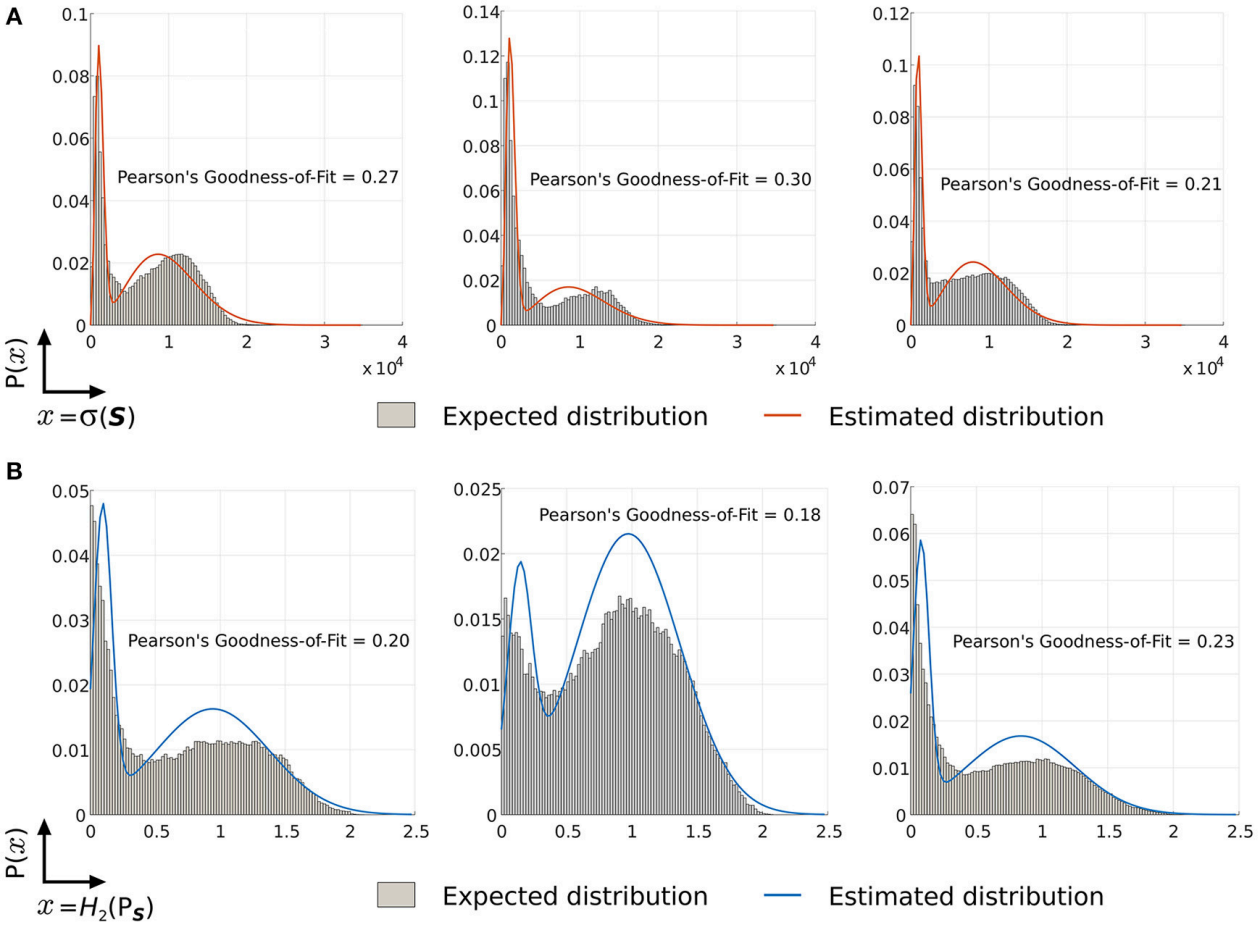
**Estimated fit mixture densities**. **(A)** Approximated density functions for standard deviation classification metric of spike-groups. **(B)** Approximated density functions for Renyi's second order entropy metric of spike-groups.

A second order Renyi's entropy with a Gaussian kernel was used for the non-parametric classification approach described in Section 2.4.1. Choosing entropy as the classification metric is equivalent to classification using information content of a random variable (Baraniuk et al., 2001). Classifying spike-groups by quantifying their information content is also equivalent to characterizing the degree of information change by micromotion profiles. Spike-groups that belong to a background category have a strong correlation to the motion profile used and, hence, exhibit less entropy (see Section 2.2). In addition, we chose a second order Renyi's entropy with a Gaussian kernel to reduce computational complexity (Principe et al., 2000; Principe, 2010). The kernel bandwidth ($h=\sqrt{2}\sigma$, see Equation 20) controls the classification accuracy of the method because it constrains the probability density function (pdf) estimation and subsequently the entropy estimation. Through our experiments, we found the kernel metric as described in Silverman (1986) and Wilcox (2012) to provide good results. Figure 12 shows approximated mixture density functions on sample data with standard deviation and Renyi's entropy discrimination metrics. Such an estimation is helpful in deciding the class posteriors, especially when more than two classes are present (more than one moving object).

#### 3.5.4. Motor control parameters

Motor control parameters affect the motion of sensor and, therefore, are important for spike-group assignments. The velocity transition levels are determined by the user and is dependent on the required average velocity of a robot's motion. From our experiments, we found velocity levels of 60–100 and 70–100 rpm with 5 cm diameter wheels to be suitable.

**Figure 13:**
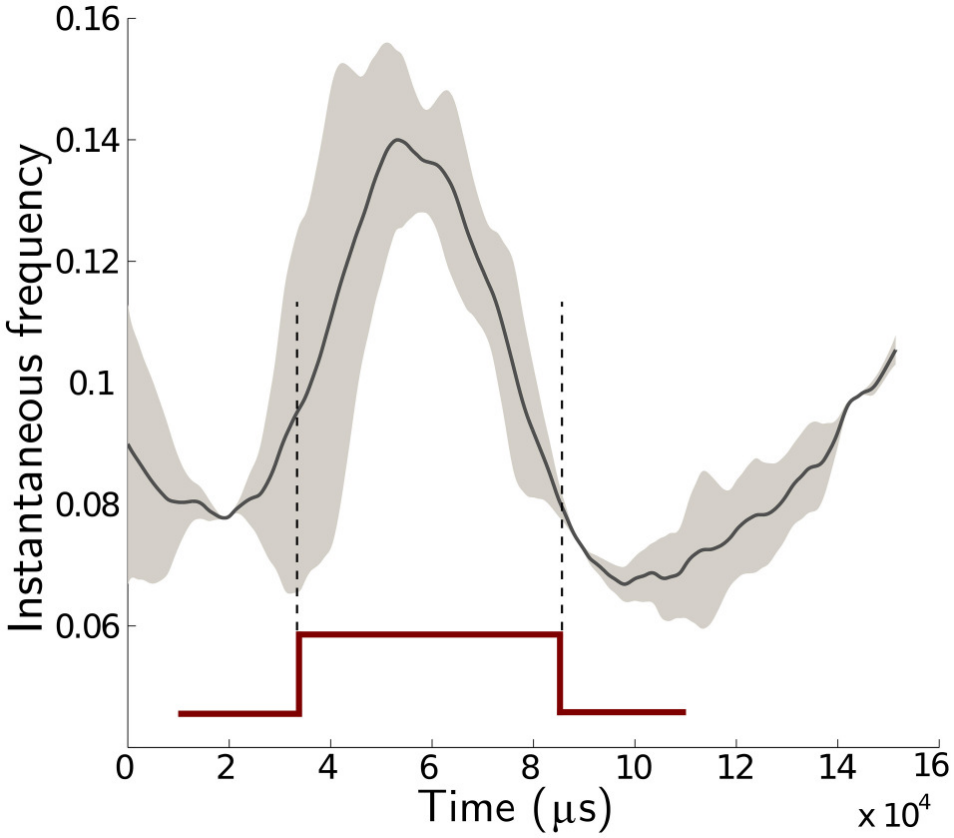
**Analysis of instantaneous frequency during a saccadic motion**. The data consists of one moving foreground object in a cluttered background scene. The solid black is the mean value of 1, 200 saccadic profiles and the gray area is one standard deviation about the mean.

The parameter *r*, is defined as

(23) $$\begin{array}{l} 2T_{a}+T_{b}=\frac{10^{6}}{f},\\ \;r=\frac{T_{a}}{T_{b}} \end{array}$$

which is the ratio of low velocity phase (*T*_*a*_) and normal velocity phase (*T*_*b*_) duration. This controls the shape of a square pulse used to perform micromotions. The discriminative power of the algorithm is a result of the enhanced statistics computed from the micromotion profiles. Since a pulse is smoothed by the spike train group definitions, (see Figure 13), the value of *r* must be long enough for the robot to be at each state for a sufficient duration. If the value of *r* increases, the robot (and mounted sensor) is at high velocity phase for less time, which results in a loss of unique distinguishing statistics. The results illustrated in Figure 9 demonstrate that a value of *r* = 0.5 coupled with saccade frequency of *f* = 10 Hz provided the best performance accuracies across dataset-2.

#### 3.5.5. Real-time implementation

The algorithm can be modified to perform in (near) real-time by replacing the *q* length spike-group array manipulations (Equations 8, 19) at each pixel to an online iterative update rule (Xu et al., 2003). Further optimization such as removing conditional executions and modulo operations are performed to obtain the fastest computational time. Moreover, the background activity filter (BAF) was turned on; hence, removing the need for parameter ρ (temporal window size). A pseudo algorithm has been provided in Supplementary Data Sheet 1.

**Figure 14:**
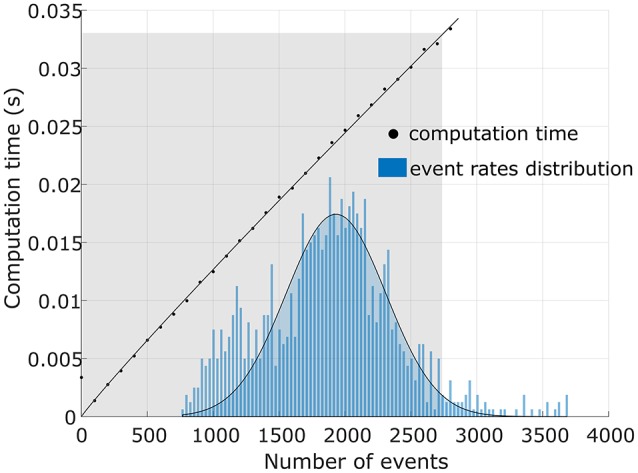
**Analysis of real-time performance**. The optimized algorithm was implemented on a 3.4*GHz*. i7 CPU machine. The plot shows that the algorithm performs in real-time zone (≥30 fps) for 98% of data.

In Figure 14 the shaded area illustrates that the algorithm is real-time for 98% of Gaussian distributed event rates (blue). Data were obtained in batches of 0.33 s (1/30 s) in a scene containing an oscillating pendulum with a cluttered background. The distribution of the number of events across these data batches is estimated as a Gaussian distribution. Finally, for consecutively increasing event numbers, computation times were recorded utilizing the optimized algorithm as depicted by a straight line in the plot. The shaded area shows the zone for real-time operation of our algorithm, which is 30 fps. This plot also shows that the time complexity of this real-time implementation is *O*(*n*).

## 4. Discussion

This paper presented the concept of utilizing a controlled sensor platform's vibrations to perform motion segmentation. The use of an event-based visual sensor was instrumental to significantly increase processing speed and accuracy. Since such sensors are analog chips, the overall power consumption is significantly reduced. We described methods for classification that used a metric F(·) for discriminating a spike-group based on its timestamp differences. The bimodality exhibited by these statistics demonstrates that moving objects impose their own statistics. These statistics are magnified and observable due to the velocity profile of the robot. The motor sensory loop described in this paper are disjoint, implying that the strategy for application of micromotions is unaffected by the scene parameters. This could be transformed to a feedback based closed motor-sensory loop where the frequency of micromotions can be altered as desired for robust classification in dynamic environments. The optimal parameters described in Table 2 will work for most scene types and robot's velocity. With more clutter in a scene, the micro-motion parameters can be increased to achieve better discrimination capability and vice versa.

If the velocity of a moving object is low, the statistical information contained in spike-groups is not able to differentiate it from a background class. The distance and projected velocity of the object also affects performance accuracy. As the distance of the moving object and robot increases the effective projected angular velocity decreases, lowering the probability of the spike-group being classified as a moving object. In Figure 7A, the algorithm performed well when the background was not very densely cluttered. However, the performance remained accurate even for the case where the background was densely cluttered. In Figure 7B, the object distance was gradually decreased with respect to the robot. This resulted in a more dense labeling of the moving object for frames closer to the sensor. In addition, if the object's projected velocity on the sensor's plane is low, accurate classification is challenging. A possible solution to these cases is to increase the micromotions frequency of the robot in order to produce more discriminative statistics.

Since spike-groups represent edges of objects, it allows our algorithm to track the exact object boundary instead of tracking an area around the object. This is important for subsequent processing of the segmented image for object recognition. Alternatively, it may be possible to use optical flow to estimate optimal spike-groups and apply the metrics in this work. A spike-group implementation approximates the continuity of events caused and estimates similarly caused events through spatial and temporal modeling. Another filtering layer on top of the motion segmentation framework can be used to provide accurate tracking results. In this paper, we have used standard deviation and Renyi's entropy as discrimination metrics for classifying spike-groups. In the case of Renyi's entropy, it is also possible to use a Gaussian mixture for clustering instead of entropy based clustering to assign clusters to spike-groups (see Figure 12).

## 5. Future work

A generalized mathematical framework for asynchronous data processing using spike-groups will be presented in a future study. This structure will be used to develop an autonomous robot tracking-following application using two robots. In addition, to aid with the tracking-following task with multiple agents, we will develop an object recognition methodology that is able to take advantage of the segmentation performed by our algorithm. Motion segmentation support will be integrated with the robot operating system (ROS) drivers for DVS and DAVIS. We are also actively developing a SNN implementation of our technique for fast parallel computations. Embodied cognition or action based perception strategies using the neuromorphic imagers for other tasks will also be explored. Lastly, we are currently performing a comparative study to test our algorithm's performance with existing neuromorphic benchmarks for tracking (Hu et al., 2016) and developing our own dataset with varying scenes and micro-motion parameters, which will be the subject of a separate study. This will be used to evaluate the applicability of the current algorithm with inherent vibratory noise in most mobile robotic systems as compared to implementing controlled micro-motions.

## 6. Conclusions

In this paper, we have presented a novel signal processing framework for asynchronous data provided by neuromorphic imagers such as the DVS. The algorithm was successfully used for motion segmentation, allowing distinction of moving objects in a scene from static background information. Motion segmentation is an important pre-processing step for many neuromorphic applications such as tracking-following and dynamic object recognition. The concept of partitioning data into redundant spike-groups stored at pixel locations allows inference of spatiotemporal features for each motion event. The process of forming spike-groups is equivalent to online clustering of spatiotemporal data into similar event classes such as dynamic objects or static background. Our algorithm introduces the concept of motion induced sensory visual perception using neuromorphic imagers, which is a common technique used in biology. This technique allows motion induced information to be captured asynchronously by the temporal data captured by a neuromorphic visual sensor. We have extended the concept of *dynamic retina* to show how temporal variations through behavioral strategies can contribute to spatiotemporal signal processing. Another significant contribution of our methodology is that it enables model free segmentation of moving objects.

## Author contributions

AM and RG developed the concept and strategies for spike-group formulation and classification. AM performed the experiments and wrote the paper in conjunction with RG, JP, and SK. Classification and distribution estimation strategies and numerical results were guided by JP, NT, and SK. Detailed refinement and fine-tuning of results and methods were guided by SK.

### Conflict of interest statement

The authors declare that the research was conducted in the absence of any commercial or financial relationships that could be construed as a potential conflict of interest.
