# Supplementary material for: A Saccade Based Framework for Real-Time Motion Segmentation Using Event Based Vision Sensors
Source: Front Neurosci. 2017 Mar 3;11:83. doi: 10.3389/fnins.2017.00083 (PMC5334512; doi:10.3389/fnins.2017.00083)
Supplement: Supplementary file 2 [file DataSheet1.pdf]

ALGORITHMS

Algorithm 1: ASSIGN SPIKE-GROUP

Data: $MeanArr^{128\times128\times p}$: To store p length spike-groups at each pixel.
$TimeArr^{128\times128}$: Time stamps of latest motion event in the spike-group at location x, y
$DelTimeArr^{128\times128}$: Temporal differences of the tail of spike-group at location x, y
ρ: Temporal threshold after which any motion event is most probably noise. Default value is $50000\mu s$.
$N(i)$: Returns the corresponding neighborhood pixel address location as x, y where $i \in (1, window)$

Input: $r, f, \mathbb{M}^t_{x,y}, window(default \leftarrow 3\times3), p(default \leftarrow 27$ number of elements in spike-groups)

Output: The spike-group to which the new motion event $\mathbb{M}^t_{x,y}$ belongs to is updated in $MeanArr$. Other outputs like $TimeArr$ and $DelTimeArr$ are also updated and returned.

$flag \leftarrow 1$
$delT \leftarrow 0$

while $flag = 1$ **do**
- $i \leftarrow 1$
- $pos \leftarrow 0$
- $H(1..window) \leftarrow \infty$
- **while** $i <= window$ **do**
  - $m = |t - TimeArr(N(i)) - DelTimeArr(N(i))|$
  - *OR*
  - $m = \left|\log \frac{(t - TimeArr(N(i))) + \epsilon}{DelTimeArr(N(i)) + \epsilon}\right|$
  - **if** $t - TimeArr(N(i)) \leq \rho$ **then**
    - $H(i) = m$
- $min\_pos = min(H)$
- **if** $min\_pos \neq \infty$ **then**
  - $flag = 0$
  - $MeanArr(x, y) = MeanArr(N(min\_pos))$
  - $MeanArr(x, y) = push(MeanArr(x, y), t - TimeArr(N(min\_pos))$
  - $TimeArr(x, y) = t$
  - $DelTimeArr(x, y) = t - TimeArr(N(min\_pos))$
- **else**
  - $TimeArr(x, y) = t$
  - $DelTimeArr(x, y) = 0$
  - $flag = 0$

return *TimeArr, DelTimeArr*
return *MeanArr*

Algorithm 1. Spike-group formation during motion events. Algorithm is provided with variables, inputs and outputs described.
